# Sequence‐specific DNA binding by AT‐hook motifs in MeCP2

**DOI:** 10.1002/1873-3468.12328

**Published:** 2016-08-09

**Authors:** Matthew J. Lyst, John Connelly, Cara Merusi, Adrian Bird

**Affiliations:** ^1^ The Wellcome Trust Centre for Cell BiologyUniversity of EdinburghEdinburghUK

**Keywords:** AT‐hook, MeCP2, Rett syndrome

## Abstract

MeCP2 is a chromatin‐associated protein that is mutated in Rett syndrome. Its methyl‐CpG‐binding domain interacts with DNA containing methylated cytosine, but other modes of recruitment to the genome have also been proposed. Here, we use *in vitro* and *in vivo* assays to investigate the DNA binding specificity of two AT‐hook motifs in MeCP2. One exhibits robust sequence‐specific DNA binding, whereas the other is a much weaker AT‐hook. Our data indicate that these motifs are secondary contributors to DNA binding by MeCP2, and this view is supported by the absence of disease‐causing missense mutations at these sites.

## Abbreviations

**MBD**, methyl‐CpG‐binding domain

**NID**, NCoR/SMRT interaction domain

**RTT**, Rett syndrome

Rett syndrome (RTT) is a severe neurological disorder caused by mutations in the X‐linked gene encoding the methyl‐CpG‐binding protein *MECP2* 1. It affects approximately 1 in 10 000 females, but is much rarer in males, where loss‐of‐function mutations give rise to a severe neonatal encephalopathy and early death. MeCP2 is a chromatin‐associated protein that contains a methyl‐CpG‐binding domain (MBD) 2, 3. Much evidence suggests that the MBD is the primary mediator of MeCP2 binding to the genome. Firstly, the MBD binds to DNA *in vitro* in a manner that is highly specific for sequences containing methylated cytosine 3, 4, 5. Secondly, chromatin immunoprecipitation analysis of MeCP2 in the brain revealed preferential binding to methylated sequences, and peaks of MeCP2 binding at sites of cytosine methylation 4, 5, 6. Thirdly, genetic ablation of DNA methylation in embryonic stem cells led to a widespread redistribution of MeCP2 bound to the genome 7. Finally, the crystal structure of the MBD bound to DNA has been solved, and this revealed the atomic details of how methylated sites are recognized 8.

The finding that deletion of the DNA methyltransferases leads to MeCP2 redistribution along the genome, rather than release from chromatin 7, suggests that DNA binding is not purely methylation‐dependent and MBD‐mediated. Indeed, other modes of MeCP2 binding to the genome have been proposed. For example, the MBD of chicken MeCP2 has been shown to interact *in vitro* with specific unmodified DNA sequences 9. Furthermore, three AT‐hook motif sequences have been proposed to exist in MeCP2 2, 10. AT‐hooks are short DNA‐binding motifs, which interact with the wide minor groove of AT‐rich sequences via the core consensus amino acid sequence RGRP 11, 12, 13. The sequence of the first of these motifs, AT‐hook 1 (amino acids 184‐195), was identified in the initial report on MeCP2 2, but is uncharacterized experimentally and of unknown functional significance. Sequences including the second, AT‐hook 2 (amino acids 264‐273), are involved in determining the clinical severity of different RTT truncation mutations 10. DNA binding by this motif has been reported, but sequence specificity has yet to be explored 10. A third motif, AT‐hook 3 (amino acids 341‐364), has also been proposed to exist in MeCP2 10. However, unlike AT‐hooks 1 and 2, this motif has not been annotated by InterPro (http://www.ebi.ac.uk/interpro/). It also contains inserted sequences, which disrupt the RGRP core motif 10, and are likely to prevent DNA binding.

In this study, we assess the DNA binding specificity of the two InterPro‐annotated MeCP2 AT‐hooks using both *in vitro* and *in vivo* techniques. We report that AT‐hook 1 shows a pronounced preference for AT‐rich DNA, whereas AT‐hook 2 has weaker AT‐hook character. Both motifs bind to AT‐rich DNA much less strongly than a consensus AT‐hook motif derived from the high mobility group HMG‐I proteins 12. In mouse cells, most residual binding of MeCP2 to AT‐rich heterochromatin, in the absence of a functional MBD, is attributable to these two motifs. In agreement with the biochemical analyses, AT‐hook 1 makes a greater contribution to *in vivo* binding than AT‐hook 2. Consistent with the AT‐hooks being minor contributors to DNA binding is the absence of mutations causing RTT‐related disorders in these motifs.

## Materials and methods

### Cell imaging

The EGFP‐MeCP2 expression plasmid has been previously described 14. The AT‐hook 1 mutation in this vector has also been described 14. Where indicated, F155S and R268Q or R270X mutations were introduced into this construct to inactivate the MBD and AT‐hook 2, respectively. NIH‐3T3 cells were seeded on coverslips in six‐well plates and transfected with JetPei (Polyplus, Strasbourg, France). After 48 h, cells were fixed for 15 min in 4% paraformaldehyde before being stained with DAPI and mounted with Prolong Gold (Life Technologies). Slides were photographed on a Leica SP5 Confocal microscope at 60× magnification and 50–130 nuclei were counted for each transfection, blind to the identity of the transfected plasmid.

### Electrophoretic mobility shift assay

Oligonucleotides encoding MeCP2 AT‐hook 1 (GTGRGRGRPKGSG), AT‐hook 2 (AEADPQAIPKKRGRKP) and the AT‐hook consensus sequence from HMG‐I (TPKRPRGRPKK) were cloned between the EcoRI and NotI sites of pGEX‐4T‐1. Recombinant proteins were expressed in *E.coli* BL21(DE3)pLysS and purified using glutathione sepharose (GE Healthcare) essentially as described elsewhere 10. A 20μL reaction mix was assembled in Electrophoretic mobility shift assay (EMSA) buffer (10 mm Tris‐HCl pH 7.5, 50 mm KCl, 0.5 mm MgCl_2_, 0.1 mm EDTA, 0.1 mg·mL^−1^ BSA, and 5% glycerol) with poly(dA‐dT) or poly(dG‐dC) (Sigma) as a competitor DNA at the indicated concentrations. Protein concentration was 40 μm for GST alone and MeCP2 AT‐hooks 1 and 2, and 1.5 μm for the HMG‐I AT‐hook. As probe, we used 0.5 ng of a ^32^P‐end‐labeled restriction fragment derived from the mouse major satellite 2. Complexes were incubated on ice for 30 min, and then resolved by electrophoresis on native 6% acrylamide gels. Competition levels were visualized by phosphorimager analysis and quantified using ImageJ (NIH, Bethesda, MD, USA). Experiments were performed in duplicate.

## Results

### Sequence‐specific DNA binding by MeCP2 AT‐hook motifs *in vitro*

**Figure 1:**
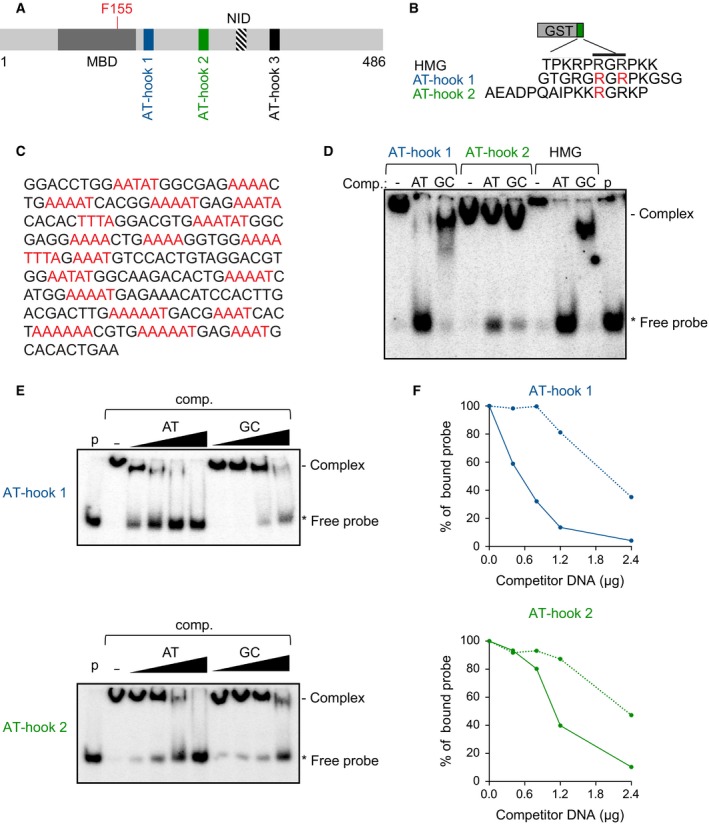
MeCP2 AT‐hooks 1 and 2 show specific affinity for AT‐rich DNA. (A) Domains and features of MeCP2. AT‐hook 1 (blue), AT‐hook 2 (green), AT‐hook 3 (black), the F155 residue (red) in the methyl‐CpG‐binding domain (MBD) (dark gray) and the NCoR/SMRT Interaction Domain (NID) (hatched box) are highlighted. (B) Fusion proteins between glutathione‐S‐transferase (GST) and AT‐hook containing peptides from HMG‐I, or MeCP2 AT‐hook 1 or 2. Arginine residues in red were mutated in the full‐length protein for imaging experiments (see Fig. 2). (C) Sequence of EMSA probe (234 base pairs) corresponding to mouse major satellite DNA. AT‐tracts >4 nucleotides in length are shown in red. (D) EMSAs showing effect of competition (comp.) by unlabeled poly(dA‐dT) (AT) or poly(dG‐dC) (GC) on protein–DNA complexes. Lane p contains probe alone. (E) Comparing the effects of varying amounts of AT or GC competitor on complexes between AT‐hook 1 or AT‐hook 2 and major satellite DNA probe. (F) Quantification of EMSA data from previous panel. Competition with poly(dA‐dT) is indicated with unbroken lines and competition with poly(dG‐dC) by dotted lines.

To better characterize the determinants of DNA binding by MeCP2, we expressed peptides corresponding to AT‐hooks 1 and 2 of MeCP2 (Fig. 1A) as GST fusion proteins (Fig. 1B) and performed EMSAs. A consensus AT‐hook motif derived from the HMG‐I proteins was also used as a comparator 12. We used a radio‐labeled probe derived from AT‐rich mouse satellite DNA (Fig. 1C), and sequence specificity was established by competition with unlabeled poly(dA‐dT) or poly(dG‐dC). Both AT‐hook 1 and AT‐hook 2 from MeCP2 were able to form complexes with the probe, but binding required approximately 25‐fold more protein than was needed with the control motif from HMG‐I (see Materials and Methods), indicating much weaker binding to DNA. Poly(dA‐dT), but not poly(dG‐dC), effectively abolished binding by both the control AT‐hook from HMG‐I, and AT‐hook 1 from MeCP2, suggesting that these peptides do indeed bind preferentially to AT‐rich DNA. Binding by AT‐hook 2, on the other hand, was only minimally inhibited by competitor DNA, and poly(dA‐dT) interfered with binding only slightly more efficiently than poly(dG‐dC), suggesting that this peptide has much poorer AT‐hook character (Fig. 1D).

To further investigate the sequence specificity of DNA binding by MeCP2 AT‐hooks 1 and 2, we performed EMSAs using a range of concentrations of poly(dA‐dT) or poly(dG‐dC) competitor DNA (Fig. 1E). The results confirmed that AT‐hook 1 is much more sensitive to competition from poly(dA‐dT) than poly(dG‐dC), whereas the differential effects of the two competitors on AT‐hook 2 were very subtle by comparison (Fig. 1F). For example, 0.8 μg of poly(dA‐dT) displaced approximately two‐thirds of the probe bound by AT‐hook 1, but only one‐fifth of the probe bound to AT‐hook 2 (Fig. 1F). On the other hand, the effects of poly(dG‐dC), a control DNA, were very similar for both AT‐hooks 1 and 2 (Fig. 1E,F). Thus, by varying competitor DNA concentrations, we were able to reveal a weak AT‐hook character of the AT‐hook 2 motif from MeCP2. Finally, we performed a control EMSA experiment using GST alone. The specificity of our assay was confirmed since this protein failed to bind to the major satellite probe DNA (Fig. S1). Collectively, our data suggest that both AT‐hook 1 and AT‐hook 2 of MeCP2 show specificity for binding to AT‐rich DNA, but both bind less efficiently than the consensus motif from HMG‐I, and furthermore, AT‐hook 2 has substantially weaker AT‐hook character than AT‐hook 1.

### Heterochromatin binding by MeCP2 AT‐hook motifs *in vivo*

**Figure 2:**
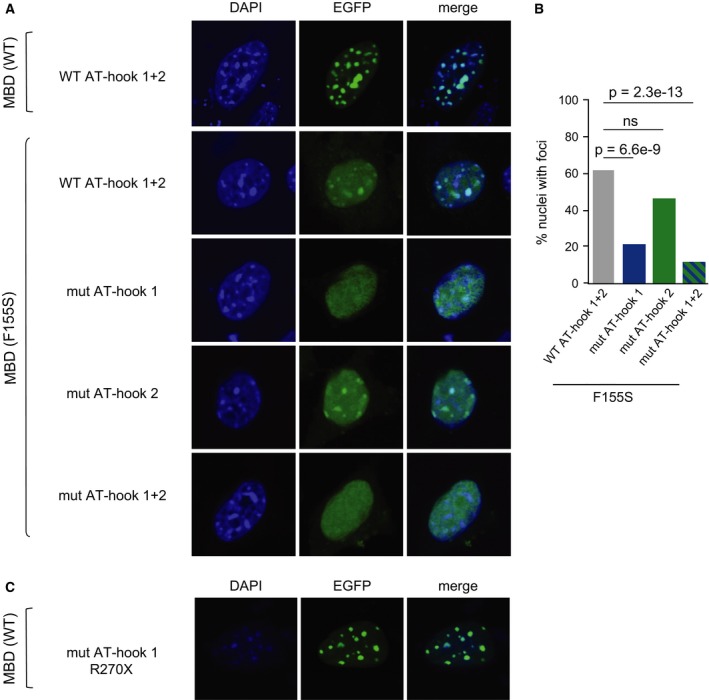
Residual chromatin binding is mediated mainly by MeCP2 AT‐hook 1. (A) EGFP‐MeCP2 was expressed in NIH‐3T3 cells and observed by fluorescence microscopy. MeCP2 was either wild‐type (WT) or carrying the MBD mutation F155S (see Fig. 1A). Mutant MeCP2 was also mutated (mut) at either AT‐hook 1 or 2 (see Fig. 1B). Panels show examples of nuclei with strong localization to foci (WT), diffuse localization with clearly visible heterochromatic foci (F155S with intact AT‐hooks 1 and 2, and F155S with mut AT‐hook 2) or diffuse localization without detectable foci (F155S with mut AT‐hook 1 and F155S with mut AT‐hooks 1 and 2). (B) Counts of F155S transfected nuclei with WT or mutant AT‐hook 1 and/or AT‐hook 2. In replicate experiments, counting was performed blind to the genotype of the transfected constructs. Indicated p‐values were calculated using Fisher's exact test. (C) EGFP‐MeCP2 with a mutation in AT‐hook 1 as well as a truncation (R270X) that abolishes AT‐hook 2 was observed in NIH‐3T3 cells. The wild‐type MBD is sufficient to localize the protein to heterochromatin.

So far, our experiments have been restricted to an *in vitro* analysis of short fragments of MeCP2. To investigate the significance of these motifs *in vivo* in the context of the full‐length protein, we developed an assay based on the expression of MeCP2 as an EGFP fusion protein in mouse NIH‐3T3 cells. In mouse cells, wild‐type MeCP2 strongly localizes to the prominent heterochromatic foci due to the presence of repetitive major satellite DNA, which is both AT‐rich and also contains approximately 40% of the 5‐methylcytosine in the genome 2. Mutation of AT‐hook 1 in the presence of a functional MBD has previously been shown to have no effect on the kinetics of chromatin binding by MeCP2 14. We therefore hypothesized that inactivating the MBD of MeCP2 would be necessary to allow us to assay any weak AT‐hook‐mediated residual binding to the major satellite. To this end, we introduced into MeCP2 the RTT‐causing F155S mutation, which is predicted to lead to unfolding and inactivation of the MBD 15. Compared to wild‐type EGFP‐MeCP2, the mutant protein produced a much more diffuse nuclear localization (Fig. 2A). However, in approximately 60% of cells, the EGFP‐MeCP2^F155S^ mutant protein still gave rise to clear foci of fluorescence coinciding with heterochromatin (Fig. 2B). To ask whether these foci were due to the AT‐hook motifs in MeCP2, we mutated these regions singly, or together, in the context of EGFP‐MeCP2^F155S^, and again assayed localization. Mutation of AT‐hook 1 reduced the number of nuclei with foci by about 65%, whereas mutation of AT‐hook 2 caused a much smaller reduction that only reached statistical significance in one replicate. Mutation of both AT‐hook motifs reduced the number of nuclei with foci by over 80% (Fig. 2B). We conclude that AT‐hook 1 mediates the majority of the residual heterochromatic localization of MeCP2 remaining after the inactivation of the MBD, with a smaller contribution by AT‐hook 2. This finding is consistent with our *in vitro* EMSA analysis of these regions of MeCP2.

Finally, we wished to examine the effect of simultaneously mutating AT‐hooks 1 and 2 on the localization of MeCP2 carrying a wild‐type MBD. Therefore, we expressed EGFP‐MeCP2 with a mutated AT‐hook 1 and with a truncation (R270X) that inactivates AT‐hook 2 10, and observed these cells by fluorescence microscopy. As assessed by this assay, the double AT‐hook mutant MeCP2 localized correctly to heterochromatic foci (Fig. 2C). This observation is consistent with the view that the AT‐hooks of MeCP2 are not primary determinants of its chromatin association.

### AT‐hook 1 and 2 mutations do not cause Rett syndrome

**Figure 3:**
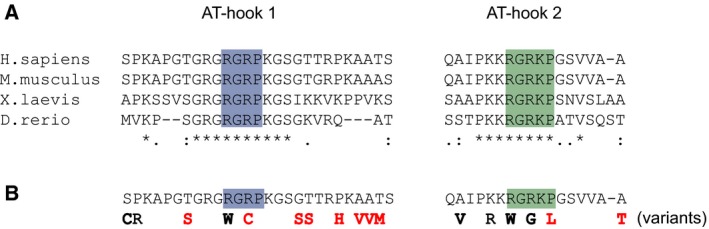
Variability at key residues of MeCP2 AT‐hooks 1 and 2. (A) Alignment of AT‐hook 1 and AT‐hook 2 motifs from MeCP2 of human, mouse, frog, and zebrafish. Residues comprising, and proximal to, the core consensus sequences of AT‐hook 1 (blue) and AT‐hook 2 (green) are highly conserved. Amino acids distal to this region show less conservation. (B) Polymorphisms in MeCP2 that affect AT‐hooks 1 and 2. Variants in male (red) or female (black) individuals were reported by the exome aggregation consortium.

MeCP2 is highly conserved among vertebrates. As well as high levels of conservation in the MBD and the NCoR/SMRT interaction domain (NID) 16, sequences comprising AT‐hooks 1 and 2 are identical between humans and zebrafish, indicating that changes are deleterious and have been selected against (Fig. 3A). In this case, mutations in the human population in these motifs may be hypothesized to be associated with disease. A mutation proximal to AT‐hook 1 has been associated with intellectual disability in males 17, but this mutation (G185V) does not affect the core RGRP motif, and is unlikely to strongly interfere with DNA binding. To investigate whether MeCP2 AT‐hook mutations are compatible with proper brain function, or whether they are associated with disease, we searched the human exome aggregation consortium dataset (http://exac.broadinstitute.org), for missense mutations that would inactivate AT‐hook 1 or 2. Males are particularly sensitive to deleterious changes in the X‐linked *MECP2* gene, with those affected by severe RTT mutations generally not surviving beyond infancy. This dataset excludes individuals with severe pediatric disorders, and so the presence of males with the loss of the second arginine of AT‐hook 1, and the proline of AT‐hook 2 (Fig. 3B), supports the view that DNA binding by these motifs is not a critical aspect of MeCP2 function. The presence of females with mutations in the first arginine of AT‐hook 1, and both arginine residues of AT‐hook 2 (Fig. 3B), is also consistent with this conclusion.

## Discussion

Our *in vitro* and *in vivo* data agree that AT‐hook 1 of MeCP2 is a robust AT‐hook, whereas AT‐hook 2 has barely detectable, AT‐hook character. In the case of HMG‐I, structural studies of two AT‐hook motifs revealed that proline residues, on each side of the RGR core motif, position the peptide backbone away from the minor groove. This allows residues surrounding the motif to make stabilizing contacts with the phosphate backbone of DNA 13, 18. We hypothesize that, together with the nature of the amino acids surrounding the core motif, the presence and positioning of proline residues might account for the weaker activity of MeCP2 AT‐hook 2 than AT‐hook 1, and also for the weaker binding of both motifs when compared with a canonical sequence from HMG‐I. In particular, AT‐hook 1 contains the core sequence RGRP but lacks an additional proline which is adjacent and N‐terminal. AT‐hook 2 (RGRKP) further lacks the proline which is immediately C‐terminal of RGR, and instead, a proline is found one more residue distal (Fig. 3A).

As a mediator of the effects of DNA methylation encoded by a gene that is mutated in a monogenic autism spectrum disorder, MeCP2 has received considerable attention. Specific disease‐causing lesions in MeCP2 are of particular interest since these allow connections to be made between RTT pathology and MeCP2 biology. Notably, RTT missense mutations primarily affect the MBD and the NID, suggesting that these are two critical interactions required for MeCP2 function 19. Outside of these domains, two disease‐causing MeCP2 truncation mutations, G273X and R270X, which differ by the presence and absence of key residues in the AT‐hook 2 motif, give rise to milder and more severe pathologies, respectively 10. These results imply that despite its weak AT‐hook character, AT‐hook 2 mediates an effect on neurological function, which is discernible in the context of a compromised truncated version of MeCP2.

It is notable that no RTT‐causing missense mutations localize to either of the MeCP2 AT‐hook motifs investigated here. Examination of the human exome aggregation consortium dataset also indicates that mutations in AT‐hooks 1 and 2 of *MECP2* do not contribute to RTT or X‐linked intellectual disability. Regions of MeCP2 that are not affected by RTT‐causing missense mutations are nevertheless highly conserved throughout the vertebrates, and this is true of the AT‐hook motifs examined in this report. This constraint over millions of years of evolution strongly implies that these regions have biological significance. What then are the physiological roles of these AT‐hook motifs in MeCP2? A plausible scenario is that AT‐hooks 1 and 2 function as secondary DNA‐binding domains, which either stabilize or modulate chromatin association by MeCP2. Alternatively, it is possible that multifaceted interactions with DNA by MeCP2 bring about chromatin compaction 10. Future biochemical and genetic studies, in particular, the creation of the appropriate cell or animal models coupled with genome‐wide chromatin immunoprecipitation analysis, promise to shed light on the molecular role of the AT‐hook motifs in MeCP2.

## Author contributions

ML, JC and AB devised the experiments and analyses used in the study. ML and CM constructed all vectors and performed fluorescence microscopy. JC designed and performed EMSA analyses. ML carried out sequence analyses. ML, JC and AB wrote the manuscript and all co‐authors passed comment.

## Supporting information


**Fig. S1.** EMSA showing that GST alone, unlike the control GST AT‐hook 2, fails to bind to the major satellite‐derived probe.
