# Supplementary material for: Sequence‐specific DNA binding by AT‐hook motifs in MeCP2
Source: FEBS Lett. 2016 Aug 9;590(17):2927–33. doi: 10.1002/1873-3468.12328 (PMC5028900; doi:10.1002/1873-3468.12328)
Supplement: Supplementary file 1 — Fig. S1. EMSA showing that GST alone, unlike the control GST AT‐hook 2, fails to bind to the major satellite‐derived probe. [file FEB2-590-2927-s001.pdf]

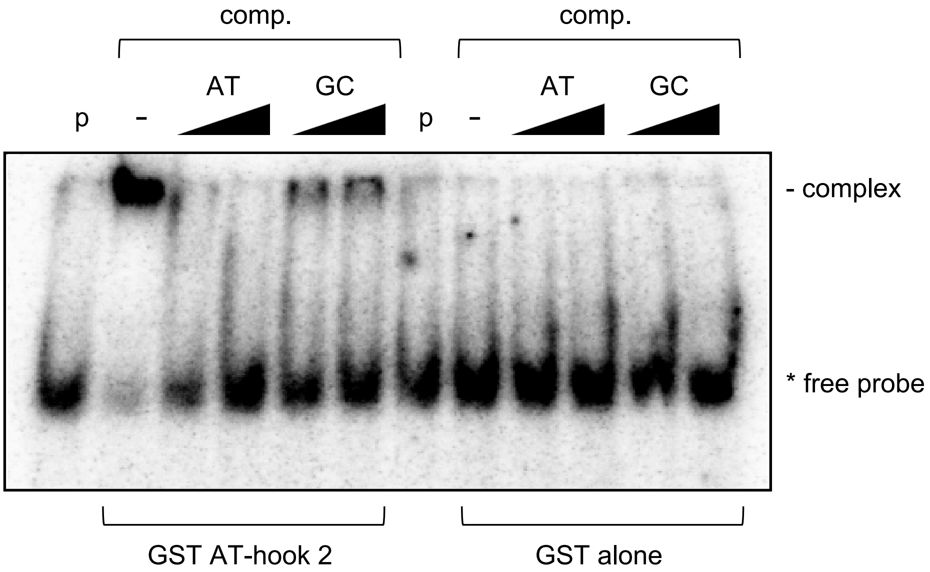

Figure S1

EMSAs showing that GST alone, unlike the control GST AT-hook 2, fails to bind to the major satellite-derived probe. Competition (comp.) is by unlabelled poly(dA-dT) (AT) or poly(dG-dC) (GC). Lane p contains probe alone.
